# Circular RNAs are associated with the resistance to Newcastle disease virus infection in duck cells

**DOI:** 10.3389/fvets.2023.1181916

**Published:** 2023-09-29

**Authors:** Lei Fan, Jinlian Ren, Yinchu Wang, Yiyi Chen, Yichun Chen, Libin Chen, Qiuyan Lin, Ming Liao, Chan Ding, Bin Xiang, Tao Ren

**Affiliations:** ^1^College of Veterinary Medicine, South China Agricultural University, Guangzhou, China; ^2^Key Laboratory of Animal Vaccine Development, Ministry of Agriculture, Guangzhou, China; ^3^National and Regional Joint Engineering Laboratory for Medicament of Zoonosis Prevention and Control, Guangzhou, China; ^4^Key Laboratory of Zoonosis Prevention and Control of Guangdong Province, Guangzhou, China; ^5^Institute of Animal Health, Guangdong Academy of Agricultural Sciences, Guangzhou, China; ^6^Shanghai Veterinary Research Institute (SHVRI), Chinese Academy of Agricultural Sciences (CAAS), Shanghai, China; ^7^College of Veterinary Medicine, Yunnan Agricultural University, Kunming, Yunnan, China

**Keywords:** waterfowl, Newcastle disease virus, duck embryo fibroblasts, circular RNAs, antiviral response

## Abstract

**Introduction:**

Newcastle disease virus (NDV) is prevalent worldwide with an extensive host range. Among birds infected with velogenic NDV strains, chickens experience high pathogenicity and mortality, whereas ducks mostly experience mild symptoms or are asymptomatic. Ducks have a unique, innate immune system hypothesized to induce antiviral responses. Circular RNAs (circRNAs) are among the most abundant and conserved eukaryotic transcripts. These participate in innate immunity and host antiviral response progression.

**Methods:**

In this study, circRNA expression profile differences post-NDV infection in duck embryo fibroblast (DEF) cells were analyzed using circRNA transcriptome sequencing. Gene Ontology (GO) and Kyoto Encyclopedia of Genes and Genomes (KEGG) enrichment analyses were used to reveal significant enrichment of differentially expressed (DE) circRNAs. The circRNA-miRNA-mRNA interaction networks were used to predict the related functions of circRNAs. Moreover, circ-FBXW7 was selected to determine its effect on NDV infection in DEFs.

**Results:**

NDV infection altered circRNA expression profiles in DEF cells, and 57 significantly differentially expressed circRNAs were identified post-NDV infection. DEF responded to NDV by forming circRNAs to regulate apoptosis-, cell growth-, and protein degradation-related pathways via GO and KEGG enrichment analyses. circRNA-miRNA-mRNA interaction networks demonstrated that DEF cells combat NDV infection by regulating cellular pathways or apoptosis through circRNA-targeted mRNAs and miRNAs. circ-FBXW7 overexpression and knockdown inhibited and promoted viral replication, respectively. DEF cells mainly regulated cell cycle alterations or altered cellular sensing to combat NDV infection.

**Conclusion:**

These results demonstrate that DEF cells exert antiviral responses by forming circRNAs, providing novel insights into waterfowl antiviral responses.

## 1. Introduction

Newcastle disease (ND), caused by the ND virus (NDV), was first identified in Indonesia in 1926 (1). ND is the most prevalent poultry infectious disease worldwide and causes severe economic losses to poultry farming industries (1). NDV infects various species, including chickens, turkeys, wild birds, and waterfowl, and undergoes cross-species transmission (2, 3). Waterfowls are natural NDV hosts or carriers but are resistant to virulent strains (4, 5). Chickens and ducks infected with the genotype VII virulent NDV strain, widespread in Asia, have variable mortality, with viruses detectable in duck organs (6, 7). Waterfowls infected with NDV strains do not exhibit overt clinical signs but can release the virus via the oropharynx or cloaca (8, 9). Ducks possess RIG-I, and with its absence in chickens, different natural immune responses to resist viral infection occur (10, 11). However, ducks exhibit resistance to NDV through unique, innate immunity, although the specific resistance mechanism remains undefined.

Circular RNA (circRNA) is eukaryotic covalently closed noncoding RNAs mainly derived from back splicing in the formation of mRNA (12). circRNAs possess a covalently closed loop without 5′ or 3′ polarities and a poly(A) tail configuration, conferring greater stability than linear RNA (13). They were originally found in plant and hepatitis viruses and considered splicing error products during mRNA formation (14). With continuous advances in high-throughput sequencing and RNA research, several circRNAs have been identified in diverse species and cell lines, and various circRNAs libraries have been supplemented (15–18). circRNA formation is a common and abundant regulation method for gene expression programs in various species; circRNAs are hypothesized to be evolutionarily conserved and show general features during gene expression (19). According to their source gene, circRNAs are classified as exonic, exon-intron, and intronic circRNAs (20).

circRNAs exert their functions by suppressing miRNA expression by functioning as specific miRNA “sponges,” acting as designated miRNA “reservoirs” to stabilize miRNA functions, modulating gene expression, and encoding proteins (20). Moreover, circRNA vaccine expressing a trimeric receptor-binding domain (RBD) of SARS-CoV-2 spike proteins could elicit potent neutralizing antibody and T-cell responses, conferring protection against SARS-CoV-2 in mice and rhesus monkeys (21). circRNAs in exosomes have been determined; their expression profiles in cancer patient sera differed from those of healthy individuals, suggesting circRNAs as possible biomarkers for cancer diagnosis (22). circRNAs participate in antiviral immune responses and viral infections affect the host circRNA expression profiles (23, 24). circRNAs also participate in antiviral responses against Ebola virus, simian virus 40 (SV40), and avian leukosis virus (25–27), indicating that circRNAs might participate in the host antiviral response.

Although circRNA expression has been detected in multiple species and cell lines, research on duck circRNAs is limited. Virulent NDV strains can infect waterfowls and chickens; however, they have different incidence and mortality rates (7). Therefore, next-generation genome sequencing was used in this study to determine and analyze circRNAs expression profiles post-NDV infection in duck cells, and enriched key circRNA functions were explored. Our results suggested that ducks might exert antiviral responses through circRNAs, providing a theoretical basis for studying waterfowl antiviral responses.

## 2. Materials and methods

### 2.1. Viruses and cells

The NDV strain rGM was preserved in our laboratory (28), multiplied in 9-day-old embryonated specific-pathogen-free (SPF) chicken eggs, and stored at −80°C. Duck embryo fibroblasts (DEF) cells were cultured and maintained in DMEM (GIBCO, Grand Island, NY, United States) supplemented with 10% FBS. Duck embryo fibroblast (DEF) cells were prepared from 9 to 10-day-old embryonated eggs as per previously described chicken embryo fibroblast (CEF) cell preparation (29).

All NDV infection related experiments were conducted in an animal biosafety level 3 facility, the protocol (SCAUABSL2022-R003) was approved by the Animal Welfare Ethics Committee of South China Agricultural University.

### 2.2. Median tissue culture infectious dose

Determination of TCID_50_ was conducted as previously described (30). DEF cells were seeded on 96-well plates with 15,000 cells in 100 μL of media per well. Plates were incubated at 37°C with 5% CO_2_. The following day, the media was aspirated and replaced by 100 μL of media containing a serial dilution of the virus (1:10–1:100). After 3 days of incubation, wells were analyzed on a standard light microscope for cytopathic effect (CPE), consisting of rounded cells, a disrupted monolayer, and clumps. The number of CPE-positive wells in each column was used to quantify the experiment by the methods of Reed-Muench (31). Briefly, DEF cells were infected with the rGM strain at a multiplicity of infection (MOI) of 1 in single cycle. The infected cultures were harvested at 4, 9, 12, and 24 h post-infection (hpi). The rGM viral titers were determined in triplicates with the standard median tissue culture infective dose assay (28).

### 2.3. Construction of circRNA library

Trizol was used to extract total RNA from DEF cells. Agarose gel electrophoresis **was** used in testing the integrity of the samples. Meanwhile, the A260/A280 ratio for the Nanodrop detection of RNA should be in the range from 1.8 to 2.0. Following rRNA depletion with Illumina Ribo Zero Gold Kit and liner RNA degradation with RNase R, circRNA was broken into short sections. A complete cDNA was obtained by addition of random hexamers, buffer, dNTPs, RNase H and DNA polymerase. To establish a whole library, the system was first configured according to the QiaQuick PCR kit protocol, then the fragment matching the length of the target gene was recovered. Finally, validation by Illumina HiSeqTM 2,500 sequencing indicated that the cricRNA library has been successfully constructed.

### 2.4. circRNA quantification and differential expression

After removing reads, including adapters, via screening raw reads obtained by high-throughput sequencing, low-quality reads with >50% useless bases and reads with more than 10% of unidentified nucleotides were removed. The remaining unfiltered reads were prepared for circRNA identification; 20mers from both ends of the unmapped reads were extracted and aligned with the reference genome to detect unique anchor positions within the splice site. Anchor reads aligning in the reversed orientation (head-to tail) indicated circRNA splicing and were then subjected to find_circ to identify circRNAs. Reads identified as circRNAs are further expanded so that the circRNA sequence inside the breakpoint of the read could be complete and surrounded by GU/AG splice sites on both sides of the breakpoint. For quantifying circRNAs, back-spliced junction reads were scaled to RPM (reads per million mapped reads), and the formula was shown as follows:


$$RPM=\frac{10^{6}C}{N}$$

Where *C* is the number of back-spliced junction reads that are uniquely aligned to a circRNA and *N* is the total number of back-spliced junction reads.

Therefore, the calculated expression can be directly used for comparing the differential expression among samples.

### 2.5. Gene ontology and Kyoto encyclopedia of genes and genomes pathway enrichment analysis

The GO and KEGG pathway enrichment analyses followed procedures described previously (32). GO enrichment analysis provided all GO terms for which source genes are significantly enriched compared to the genomic background and filtered source genes corresponding to biological functions. First, all source genes were mapped to GO terms in the Gene Ontology database; gene numbers were calculated for every term, and the significant GO enrichment for GO items compared to the reference genome was analyzed using the hypergeometric test. The formula for calculating the *p*-value is as follows:


$$P=1-\sum_{i=0}^{M-1}\frac{\left(\begin{array}{c} M\\ i \end{array}\right)\left(\frac{N-M}{n-i}\right)}{\left(\begin{array}{c} N\\ n \end{array}\right)}$$

Here *N* is the number of all genes with GO/KEGG annotation; *n* is the number of source genes in *N*; *M* is the number of all genes that are annotated to the certain GO/KEGG terms.

KEGG pathway enrichment analysis was performed with reference to the GO enrichment analysis method. Pathway enrichment analysis identified significantly enriched metabolic pathways or signal transduction pathways in source genes compared with the entire genome background. The calculation formula is the same as that in the GO analysis.

### 2.6. miRNA sponge analysis and integrated analysis of circRNAs-miRNAs-mRNAs

For circRNAs (samples collected at 9 h) annotated in circBase, the target relationship with miRNAs can be predicted using StarBase (version 2.0). Mireap, Miranda (version 3.3a), and TargetScan (version 7.0) were used to predict sample target genes for novel circRNAs. To predict mRNAs interacting with circRNAs and miRNAs, miRTarBase (v6.1) was used to predict mRNAs targeted by the miRNAs sponge. The resulting correlation of circRNAs-miRNAs-mRNAs can be visualized by Cytoscape.

### 2.7. Western blot and RT-qPCR

DEF cells were infected at 1 MOI to examine the expression of NDV nucleocapsid protein (NP). Cells harvested at the indicated time points were washed three times with PBS and lysed with RIPA buffer. Proteins were separated under denaturing conditions in 10% sodium dodecyl sulfate (SDS, Beyotime, China) polyacrylamide gels using a minigel system, then transferred to nitrocellulose (NC) membranes. The membrane was blocked for 1 h with 5% skimmed milk powder before being incubated with anti-NP antibodies (NP antibody was preserved in our laboratory) overnight at 4°C. After washing three times with PBS-Tween and once with PBS, blots were incubated with peroxidase (POD) labeled species-specific anti-immune globulin G (IgG) conjugates for 1 h at room temperature. After washing four times as mentioned above, the NC membrane was scanned and photographed by the Odyssey infrared imaging system.

### 2.8. Nucleoplasm isolation and RNA extraction

Samples were subjected to nucleocytoplasmic separation according to the instructions. RNA was extracted from cells using Trizol reagent.

### 2.9. Detection of circular RNA expression via relative quantitative RT-qPCR

RNA (1,000 ng) was used as a template, and Reverse Transcriptase M-MLV reverse transcriptase (TaKaRa, Dalian, China), Random Primers(TaKaRa, Dalian, China) and other reagents were added. Samples were kept in a 42°C constant-temperature water bath for 10 min and inactivated for 2 min at 95°C, cDNA was obtained and stored at −20°C. Primers were designed according to the serial number of the GenBank duck source gene (Table 1). Divergent primers were designed based on the circRNA predicted sequences provided by the transcriptome. Amplification was performed with ChamQ Universal SYBR qPCR Master Mix (Vazyme, Nanjing, China) by RT-PCR. After the reaction, transcription level was calculated by formula 2-(ΔΔct), and then the values were drawn into a histogram by GraphPad Prism 8 software (GraphPad Software, Inc., United States), and a difference analysis was performed.

**Table 1:** Primers used in this study.

Primer	Sequence (5′ → 3′)	Length of product
ACTB-F	CCCCATTGAACACGGTATTGTC	199 bp
ACTB-R	GGCTACATACATGGCTGGGG	
con-000094-F	GGGAGGAATGGGGAGATAGG	299 bp
con-000094-R	GTGATGACTGGTGAACGGGC	
div-000094-F	GGACTTGCCCGTTCACC	158 bp
div-000094-R	TTCGTCGCCTCTTGCTG	
con-000912-F	TGTTGCTTTGCTTGTGACTCTG	118 bp
con-000912-R	TTAAGTCCATGCGGGTTCTGA	
div-000912-F	CACAGTTTCTTGCTGACACAGAGA	119 bp
div-000912-R	GCAAACAGCAAGGCTTCCCA	
con-001672-F	AAGATGCCCAACGTCTTCCA	113 bp
con-001672-R	TGGAGTATGTCGAGGGCTGA	
div-001672-F	GCCTCCACACATTGACTATT	100 bp
div-001672-R	CAACTCTCTTTCCATCTCGT	
con-000030-F	TCAAGTCGTCCTAGCCCAGT	345 bp
con-000030-R	AGAGCAGCACAGCTACAAGG	
div-000030-F	CTTCAGGAAGAGATGGCCTGG	155 bp
div-000030-R	TGTTGTGTCACCCACGTAGC	
con-000991-F	TCCTTCGTTTACAGCCGGTC	337 bp
con-000991-R	AGAGCACACCTCTCTGGAGT	
div-000991-F	TAAGACCACCCGCGCTAGAA	147 bp
div-000991-R	GTTAGCTGGCTTGTTGGTGT	

### 2.10. Sanger sequencing (circRNA)

The reverse-spliced region of circRNA was amplified using divergent primers. The products recovered were sent to Sangon Biotech for Sanger sequencing, and the sequencing results were aligned by NCBI Blast analysis, MegAlign, and SnapGene.

### 2.11. siRNA interference test

For transfection, cells cultured in 6-well plates were washed 3 times with PBS and transfected with siRNA (Table 2) using Lipofectamine 2000 (Invitrogen) according to the manufacturer’s protocol.

**Table 2:** SiRNAs for transfection.

siRNA	Sequence (5′ → 3′)
si-circFBXW7-1 sense	CACAAAGACAAAGAAUGUGTT
si-circFBXW7-1 antisense	CACAUUCUUUGUCUUUGUGTT
si-circFBXW7-2 sense	CAAAGACAAAGAAUGUGAATT
si-circFBXW7-2 antisense	UUCACAUUCUUUGUCUUUGTT
si-circFBXW7-3 sense	GACAAAGAAUGUGAAAGCATT
si-circFBXW7-3 antisense	UGCUUUCACAUUCUUUGUCTT
NC sense	UUCUCCGAACGUGUCACGUTT
NC antisense	ACGUGACACGUUCGGAGAATT

### 2.12. Statistical analysis

Data presentation and statistical analyses were performed using GraphPad Prism (version 5.0; GraphPad Software, Inc., La Jolla, CA, United States). Data were expressed as mean ± standard deviation (SD). Data were analyzed using Student *t*-test for pairwise comparisons or an analysis of variance/Dunn multiple comparison test for multiple comparisons. Statistical significance was set at *p* < 0.05, *p* < 0.01, and *p* < 0.001 for values that were significant, very significant, and highly significant, respectively.

## 3. Results

### 3.1. Characterization of the circRNA expression profile post-NDV infection in DEFs

**Figure 1:**
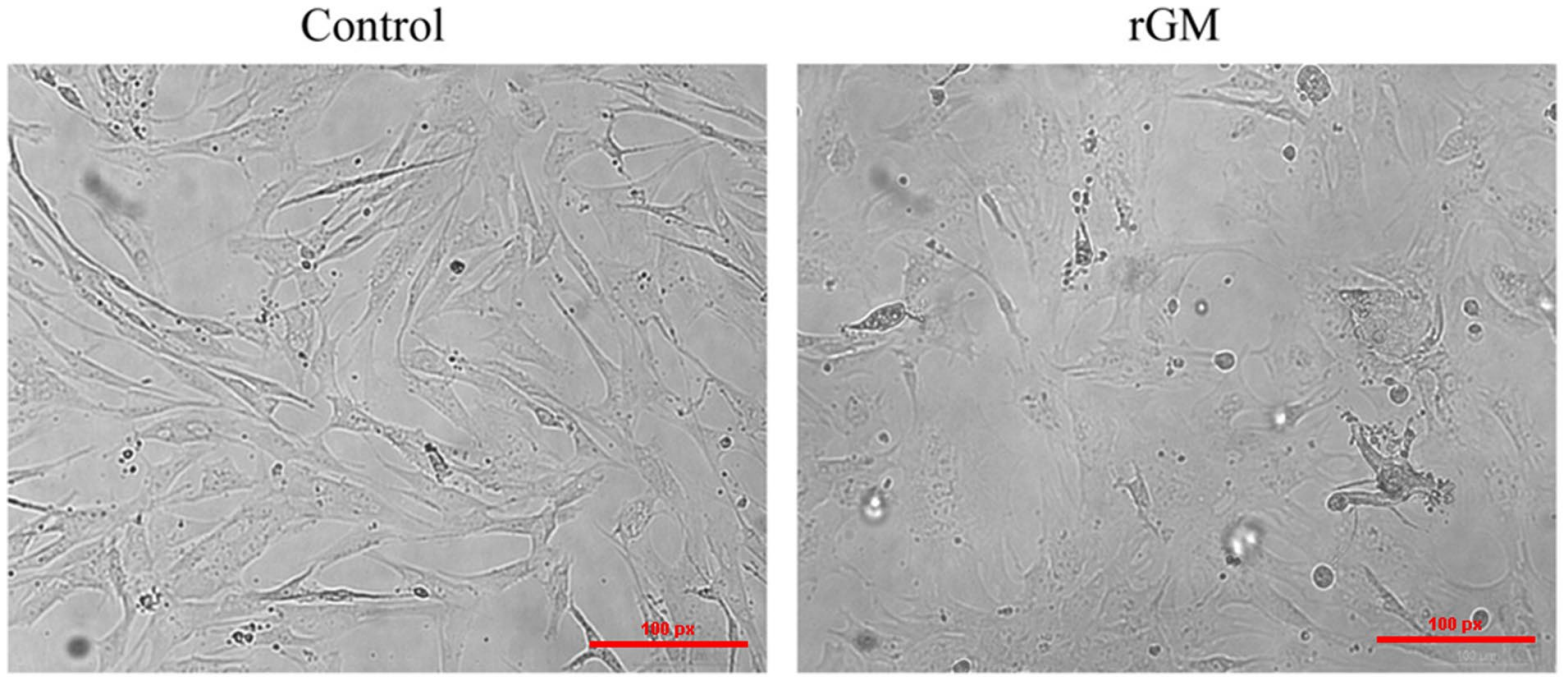
Duck embryo fibroblast (DEF) cell lesions infected with Newcastle disease virus (NDV) (100 μm). Control: DEF cells were seeded for 9 h and micrographed; rGM: DEF cells were inoculated with the NDV rGM strain for 9 h and micrographed.

**Figure 2:**
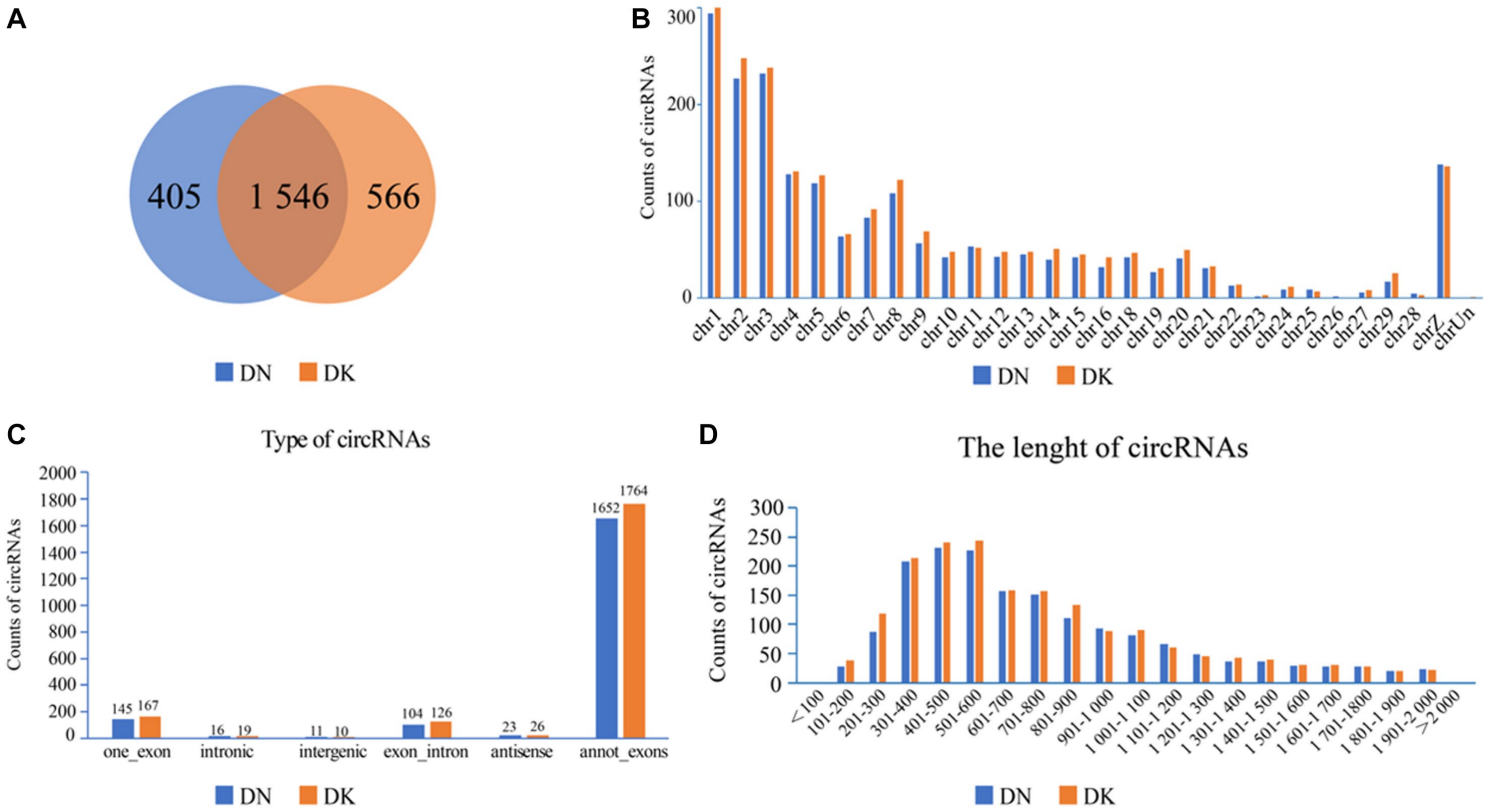
circRNA expression profile in duck embryo fibroblast (DEF) cells. **(A)** Expressed circRNAs in DEF cells with or without NDV infection. **(B)** Expressed circRNAs on each chromosome in DEF cells. **(C)** The different circRNAs expressed in DEF cells. **(D)** The length of expressed circRNAs in DEF cells.

The expression of NDV NP protein was detected by Western Blot and indirect immunofluorescence assay (Supplementary Figure S1), indicating that NDV was able to infect duck embryo fibroblast (DEF) cells. The indirect immunofluorescence assay was performed according to the method of Sun et al. (33). Lesions appeared after infecting DEF cells with NDVs for 9 h with 1 multiplicity of infection (MOI) (Figure 1); thus, 9 h post-NDV infection was selected as the transcriptome sequencing time point. Three replicate cell samples were selected from the control and experimental groups for sequencing. The control groups (DEF cells without infection of NDV) were DN-1, DN-2, and DN-3, while the experimental groups (DEF cells 9 h post-NDV infection) were DK-1, DK-2, and DK-3. The samples were subjected to transcriptome sequencing and purified to filter out the data, and 2,517 circRNAs were identified in the DEF cells. The number of circRNA intersections between the control and infection group was 1,546 (Figure 2A). Candidate circular RNAs were similar to the control group in length, chromosomal distribution, and type following NDV infection in DEF cells. These candidate circRNAs were mainly located in the exon region; the fewest circRNAs were mapped to the intergenic exon regions in DEF cells, respectively (Figure 2C). circRNAs of length 401–500 bp and 1,801–1,900 bp were the highest and the lowest, respectively, in DEF cells, and no circRNAs had a length of < 100 bp and no more than 2,000 bp (Figure 2D). The results of circRNA chromosomal distribution in DEF cells showed that most circRNAs were distributed on chromosome 1, followed by chromosomes 2 and 3; no circRNA was distributed on chromosome 17; > 50% of circRNAs were distributed on chromosome 1–5 (Figure 2B). Overall, circRNAs were identified in DEF cells, and NDV infection could affect their expression in DEF cells.

### 3.2. Identification and verification of significantly DE circRNAs

To investigate the antiviral response to NDVs in DEF cells, the circRNA expression profile was analyzed and significantly differentially expressed circRNAs (fold change >2 and *p*-value <0.05) were screened; a total of 57 (23 upregulated and 34 downregulated) genes were identified (Supplementary Table S1). Significantly differentially expressed circRNAs were identified in the DN group (non-NDV-infected DEF cells) vs. the DK group (NDV-infected DEF cells). These results demonstrate that circRNAs expressed in DEF cells show significant variations post-NDV infection.

**Figure 3:**
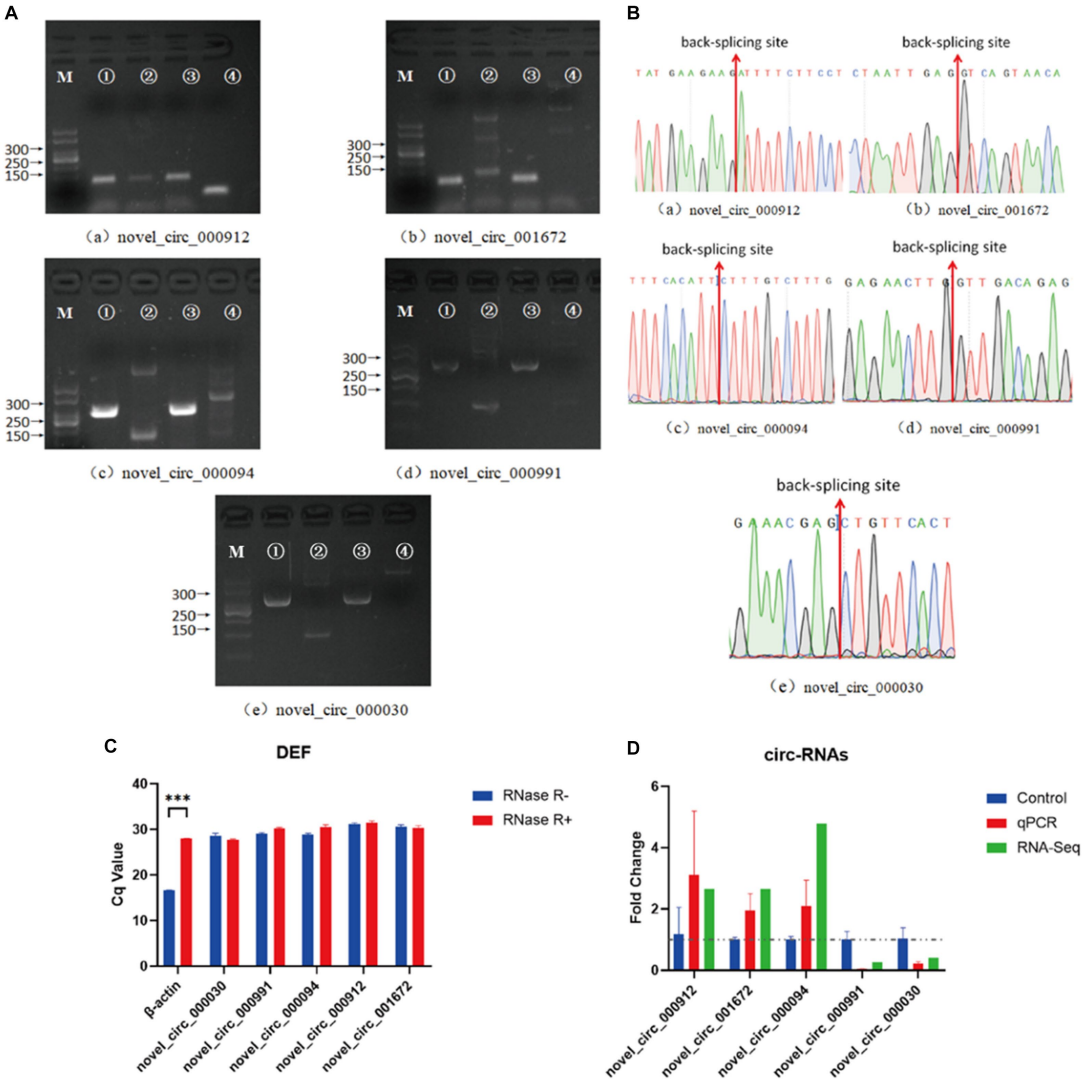
Verification of five significantly differentially expressed circRNAs in the NDV-infected DEF cell group. **(A)** Electrophoretogram of the PCR products. M: DNA marker 500; 1: Convergent primers were used for amplification using cDNA as template; 2: Divergent primers were used for amplification using cDNA as template; 3: Convergent primers were used for amplification using gDNA as template; 4: Divergent primers were used for amplification using gDNA as template. **(B)** The head-to-tail junctions of significantly differentially expressed circRNAs were confirmed via Sanger sequencing. The red line indicates back-splicing site position. The area from left (or right) of the site is the end (or start) sequence of the circRNAs (junction). **(C)** qRT-PCR was used to detect significantly differentially expressed circRNA resistance to RNase R digestion. β-actin was used as a linearity control. Data are expressed as means ± SEM (*n* = 3). **(D)** Validation of significantly differentially expressed circRNAs in the NDV-infected DEF cell group using qRT-PCR. Data are expressed as means ± SEM (*n* = 3).

In the NDV-infected DEF cell group, five significantly differentially expressed circRNAs, including three upregulated (novel_circ_000094, novel_circ_001672, and novel_circ_000912) and two downregulated (novel_circ_000991 and novel_circ_000030) circRNAs, were validated (Table 3) using reverse transcription PCR analysis, Sanger sequencing, and RNase R digestion tests. We designed converging and diverging primers to validate circRNAs; the specific sites differed and so did the size of the PCR products. Reverse transcription PCR analysis demonstrated that the fragment of interest could not be amplified using divergent primers with gDNA as a template (Figure 3A, lane 4), whereas the other lanes contained the amplified band of interest (Figure 3A, lanes 1–3), suggesting that circRNAs were circular structures derived from post transcriptional mRNA precursors via alternative splicing. Sanger sequencing of the products after amplification with divergent primers using cDNA as the template revealed that the sequencing results were consistent with the back splicing region alignment (Figure 3B), further indicating that the 5 circRNAs selected for validation were reliable. Furthermore, qRT-PCR validation using RNase R treated RNA showed that the CQ value of β-actin was significantly higher than that of the untreated group, indicating that the amount of housekeeping gene mRNA was significantly reduced following RNase R treatment, whereas the circRNAs did not change significantly after RNase R digestion (Figure 3C), indicating that the five selected circRNAs were all RNase R enzyme tolerant with stable circular structures. The qRT-PCR results indicated that the three upregulated and two downregulated circRNAs were consistent with the transcriptome sequencing results in NDV-infected DEF cells (Figure 3D). These results confirmed circRNA identification in this study as accurate and reliable.

**Table 3:** circRNAs selected for expression verification.

circRNA	Host source gene	Length	Type	*p*-value	丨log2FC丨	Up/down-regulation
novel_circ_000912	CUL1 (ncbi_101793264)	937 bp	annot_exons	0.04	18.57	Up
novel_circ_001672	NLK (ncbi_101804891)	221 bp	exon_intron	0.04	18.5	Up
novel_circ_000094	FBXW7 (ncbi_101799234)	558 bp	one_exon	0.03	2.26	Up
novel_circ_000991	BRF1 (ncbi_101796246)	539 bp	annot_exons	0.03	1.88	Down
novel_circ_000030	ETNK1 (ncbi_101801722)	544 bp	annot_exons	0.03	1.26	Down

### 3.3. KO and KEGG enrichment analyses of significantly DE circRNA

**Figure 4:**
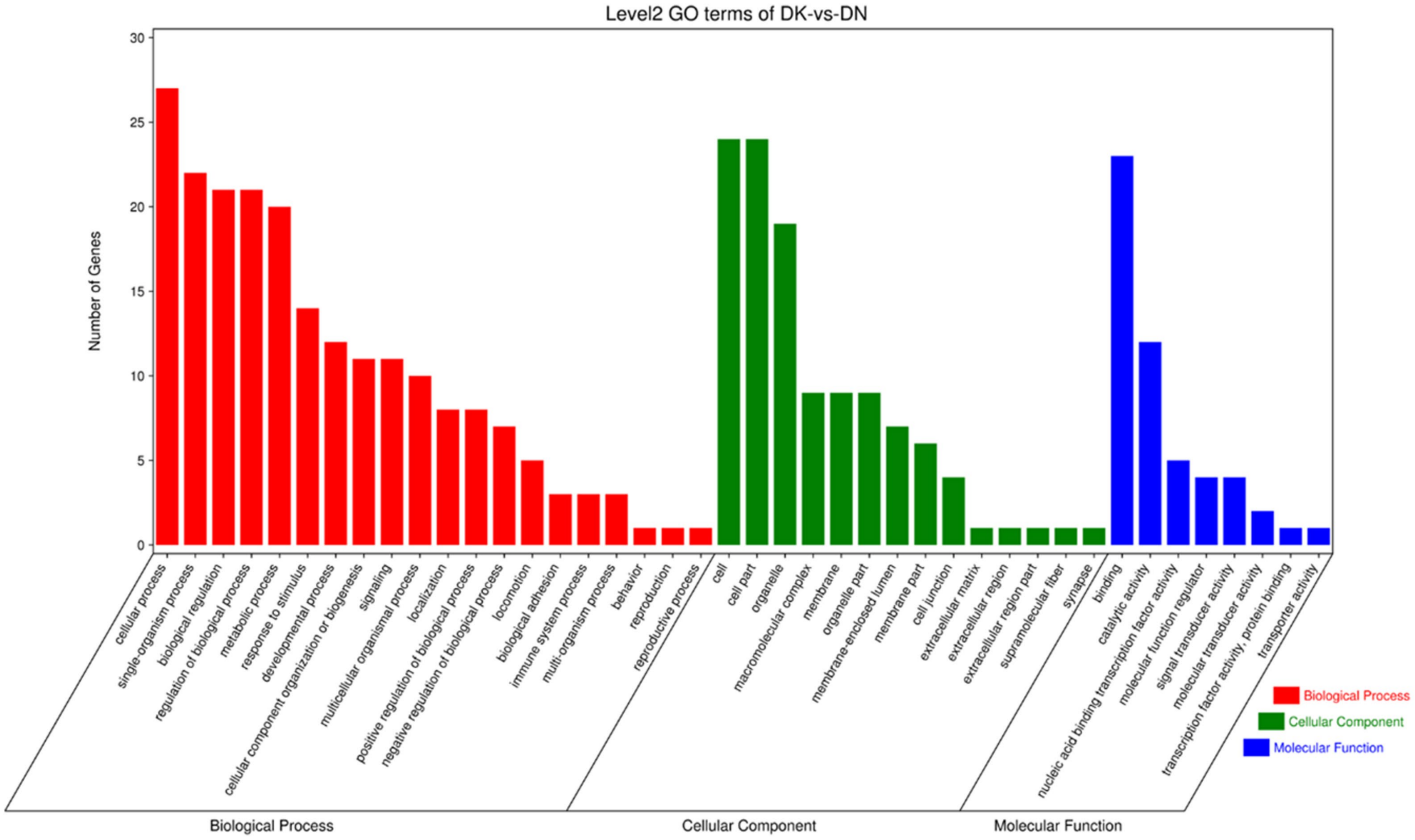
Gene ontology (GO) annotation of significantly differentially expressed circRNA from host source genes and Newcastle disease virus (NDV)-infected duck embryo fibroblast (DEF) cells.

To further explore the functions of significantly differentially expressed circRNAs produced post-NDV infection in DEF cells, the host source genes of these circRNAs were used for GO and KEGG enrichment analyses. For GO-molecular function (MF), GO-cellular component (CC), and GO-biological process (BP), the significantly differentially expressed circRNAs were mainly associated with binding and catalytic activity, cell, cell part, and organelle, and cellular, single-organism, and biological processes, respectively, post-NDV infection in DEF cells (Figure 4). GO enrichment analysis indicated that the circRNAs in DEF cells might respond to NDV infection by regulating different gene functions.

**Figure 5:**
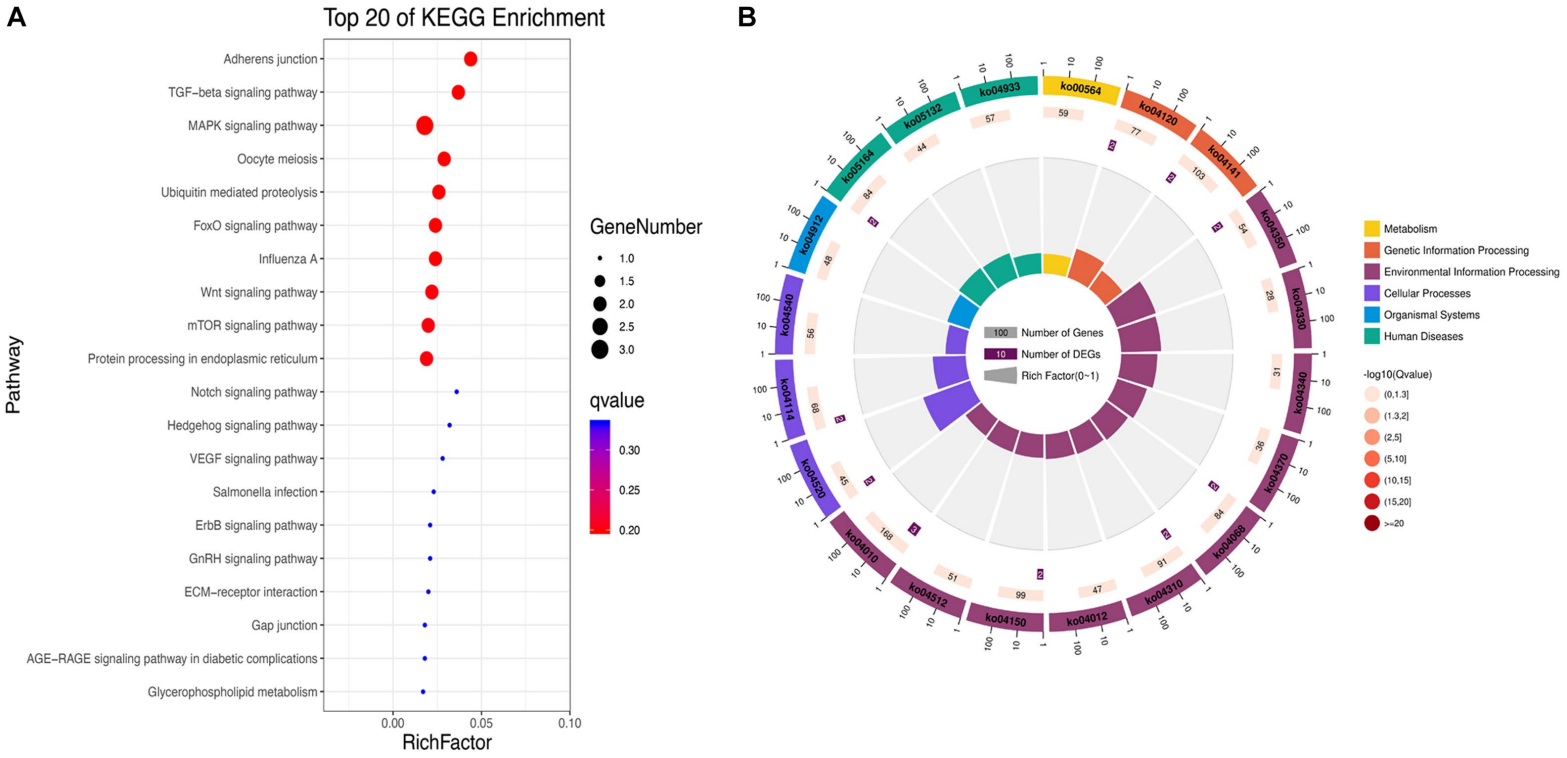
Kyoto Encyclopedia of Genes and Genomes (KEGG) pathway annotation of significantly differentially expressed circRNA. **(A)** Gradient map for KEGG pathway annotation of significantly differentially expressed circRNAs from Newcastle disease virus (NDV)-infected duck embryo fibroblast (DEF) cells. **(B)** Circular map for KEGG pathway annotation of significantly differentially expressed circRNAs from NDV-infected DEF cells.

KEGG enrichment analysis showed that the significantly differentially expressed circRNAs in NDV-infected DEF cells were mainly enriched in the FoxO, MAPK, and mTOR signaling pathways, ubiquitin-mediated proteolysis, and adherens junction (Figure 5A). Additionally, circRNAs were mainly enriched in environmental information processing-related pathways (Figure 5B). After NDV infection of DEF cells, circRNA source genes were mainly involved in regulating relevant central pathways in apoptosis-related pathways (FoxO and mTOR signaling), cell growth pathways (FoxO signaling), and protein degradation-related pathways (ubiquitin-mediated proteolysis), suggesting that circRNAs in DEF cells might respond to NDV infection by affecting signal pathways to relevant immune responses. Conclusively, after NDV infection, DEF cells may mediate the molecular mechanism to activate antiviral responses. In the NDV-infected DEF cell group, the host source genes of circRNA novel_circ_001672 and novel_circ_000094, extensively involved in multiple regulatory processes, were the most important.

### 3.4. Establishment of circRNA-miRNA-mRNA interaction networks

**Figure 6:**
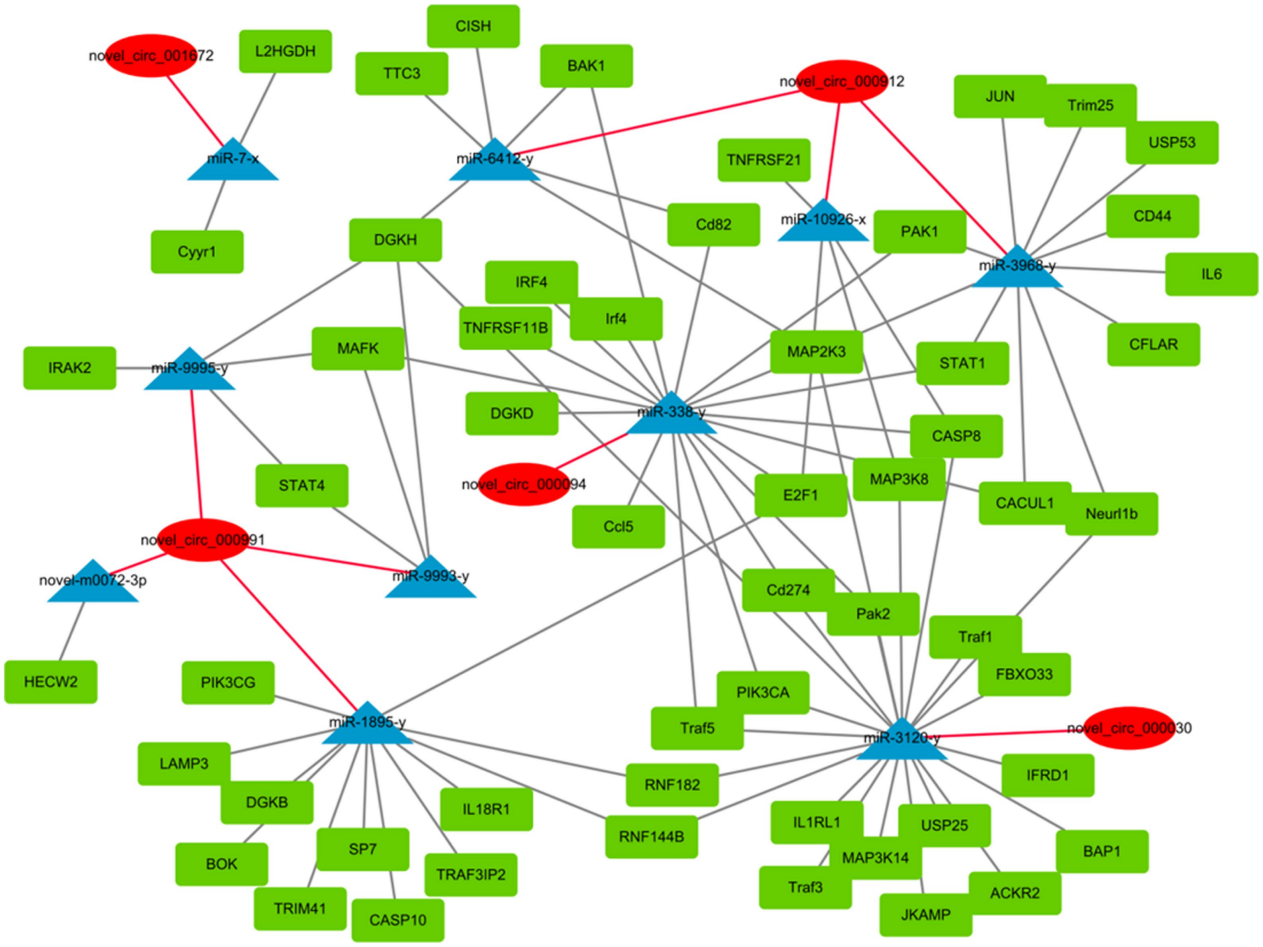
circRNA-miRNA-mRNA interaction network of significantly differentially expressed circRNAs from NDV-infected DEF cells.

circRNAs function as miRNA sponges and inhibit target gene degradation by miRNAs mediating different biological processes during virus-induced cancer and infection (25, 34). In total, 106 bound miRNAs were predicted in significantly differentially expressed circRNAs of DEF cells with NDV infection. The miRNA genes binding mRNAs were predicted to construct circRNA-miRNA-mRNA interaction networks; DEF cells targeted multiple genes related to the central pathway or apoptosis, including CASP8, BAK1, PIK3CA, MAPL3K14, and TRAF1 (Figure 6). These results suggest that DEF cells combat NDV infection by regulating cellular pathways or apoptosis through circRNA-targeted mRNAs and miRNAs. Among these, the above-mentioned mRNAs, such as CASP8, BAK1 and PIK3CA, were mainly targeted by mir-338-y, while novel_circ_000094 targeted mir-338-y, indicating a novel_ circ_ 000094 that might have potential to regulate relevant antiviral immune processes.

### 3.5. circRNA-FBXW7 affected infection in NDV-infected DEF cells

**Figure 7:**
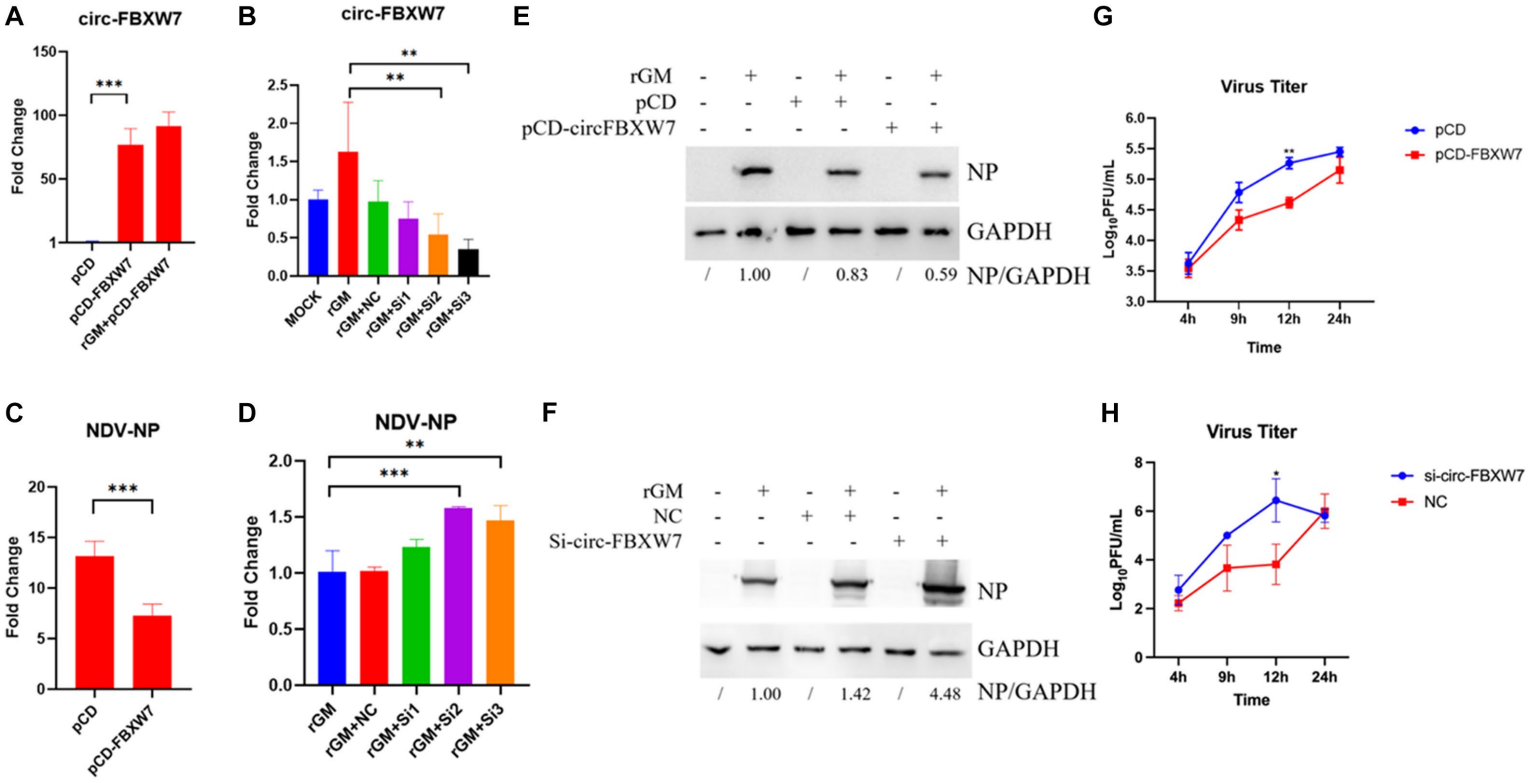
Newcastle disease virus (NDV) proliferation after circ-FBXW7 overexpression or knockdown. **(A)** qRT-PCR verification of circ-FBXW7 overexpressed plasmid expression. **(B)** qRT-PCR verification of the siRNA knockdown efficiency for circ-FBXW7. **(C)** NP gene production post-NDV infection with circ-FBXW7 overexpression determined via qRT-PCR. **(D)** NP gene expression post-NDV infection with circ-FBXW7 knockdown. **(E)** NP protein production post-NDV infection with circ-FBXW7 overexpression. **(F)** NP protein production post-NDV infection with circ-FBXW7 knockdown. **(G)** Virus growth kinetics of NDV with circ-FBXW7 overexpression. The NDV rGM strain was inoculated after transfection with the pCD-FBXW7 plasmid for 24 h in DEF cells. **(H)** Virus growth kinetics of NDV with circ-FBXW7 knockdown.

The previous enrichment and interaction network results revealed that novel_circ_000094 (circ-FBXW7) regulated multiple pathways and functional genes during NDV infection. The validation results for these genes were consistent with the sequencing results. Therefore, this circRNA was selected to determine whether DEF cells resisted NDV infection through it. Plasmids and siRNAs targeting circ-FBXW7 were constructed and designed, respectively (Figures 7A,B). And we found that si-3 for the circ-FBXW7 knockdown effect is the highest, and the following experiments were carried out using this siRNA. The effect of NDV proliferation after circ-FBXW7 knockdown or overexpression was investigated; western blot and qRT-PCR results indicated that circ-FBXW7 overexpression and knockdown suppressed (Figures 7C,E) and promoted (Figures 7D,F) NDV NP (Nuclear protein) protein expression and gene transcription levels. NDV viral growth curves showed similar results, with significantly lower and higher viral titers at 12 h post-infection after circ-FBXW7 overexpression and knockdown, respectively (Figures 7G,H). These results were consistent with the transcriptome sequencing results, suggesting that DEF cells combat NDV infection by circ-FBXW7.

### 3.6. KEGG enrichment analyses of circ-FBXW7-targeted mRNAs

**Figure 8:**
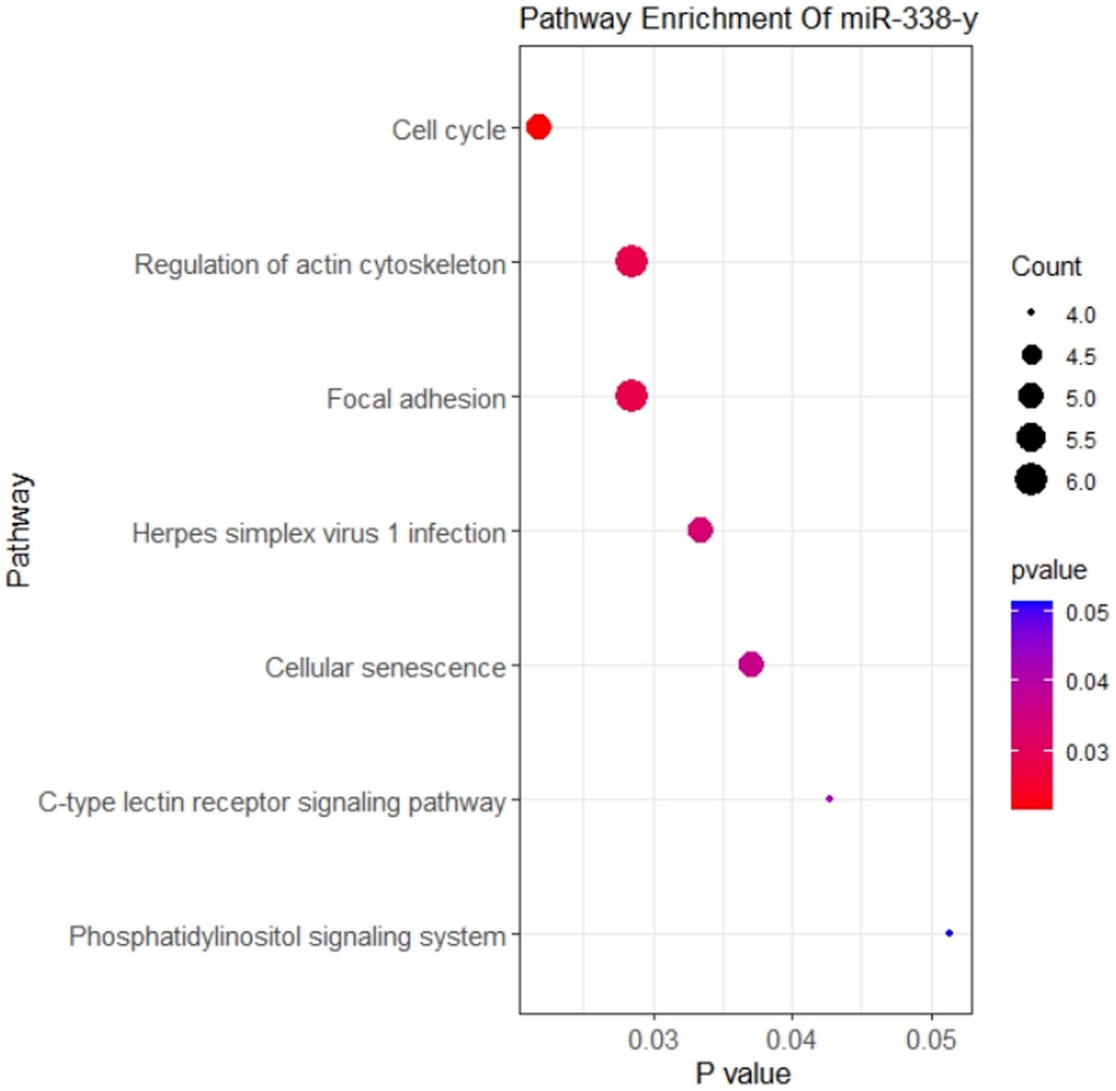
KEGG enrichment analysis of mRNA targeted by miRNA binding with circ-FBXW7.

Our study had demonstrated that circ-FBXW7 promoted resistance to NDV infection in DEF cells. Enrichment analysis of mRNAs targeted by miRNAs predicted to bind circ-FBXW7 was performed. Circ-FBXW7 was predicted to bind mir-338-y, targeting 140 mRNAs. These 140 target genes were subjected to KEGG enrichment analysis, which showed that targeted mRNAs were mainly enriched in the cell cycle and senescence signaling pathways (Figure 8), indicating that DEF cells might combat NDV infection by forming circ-FBXW7 combined with miRNAs targeting mRNAs to regulate different process.

## 4. Discussion

Genotype VII NDV strains are widespread in China (2). The NDV host range is wide and includes over 250 avian domestic and wild species (34). Waterfowls are natural NDV hosts or carriers but resist NDV infection (35). Continuous outbreaks of genotype VII NDV-induced ND in waterfowl have occurred since 1997 (36). Elbestawy et al. reported that the morbidity, mortality and viral shedding rates of chickens and ducks infected with VII NDV differ (9). Barber et al. reported a variation between chicken and duck innate immune responses, which exerted a more efficient interferon (IFN) response against viral infection via RIG-I (absent in chickens) (37), possibly contributing to the differences in susceptibility to NDV and the different clinical manifestations post-NDV infection between chickens and ducks. Several studies have shown that chickens and waterfowls combat NDV infection differently.

As covalently closed noncoding RNA, circRNAs are characterized by an abundant expression, stable structure, and evolutionary conservation (12). circRNA has been identified in several species (14–18); therefore, their expression profiles in birds, including chickens and ducks (DEF cells), are similar. Several studies have reported circRNA expression profiles in chicken, and the results were similar to that of DEF cells in this study. In a duck study by Wu et al., circRNAs in duck granulosa cells were mainly located in exons, whereas circRNAs from duck preadipocytes in a study by Wang et al. were mainly sense overlapping (38, 39), indicating that the circRNA expression profiles in ducks and other birds were relatively conservative, and their length and circRNA source chromosomes were similar to those of other birds; however, the circRNAs formed differed. The circRNAs changes in this study might be due to the mechanism by which DEF cells respond to NDV infection by forming different circRNAs to resist NDV invasion.

GO and KEGG pathway enrichment analyses of circRNA source genes are essential for exploring and predicting circRNA roles in NDV infection. Several GO annotation analyses of significantly differentially expressed circRNAs produced post-infection with different viruses revealed that the host participated in the physiological activities of various cells through circRNAs production (40–42). Consistent with our results, GO annotation analysis revealed that significantly differentially expressed circRNAs produced post-NDV infection in DEF cells are abundantly expressed in various molecular-, biological-, and cellular-related functions, indicating that circRNAs in DEF cells might broadly involve in various physiological activities of cells in response to NDV infection. Guo et al. found that chickens regulate the innate immune response to produce antibodies via circRNA with LaSota infection (43). These results indicate that infection with NDV could also affect innate immune response *in vivo*. However, Guo et al. used lentogenic strain in their study, while we used virulent strain. There might be some differences in the regulation of innate immune response between virulent and lentogenic strain. Coupled with our KEGG enrichment results, we speculated that DEF cells might affect cell-related signaling pathways for antiviral responses to NDV infection by regulating circRNA-induced ubiquitination progress. Hosts target the HCV NS5A protein via ISG12a to mediate NS5A degradation via the ubiquitination-dependent proteasome pathway for antiviral responses (44). Consistent with our KEGG enrichment results, DEF cells may utilize circRNAs to target the NDV protein degradation directly or indirectly via the ubiquitination-dependent proteasome degradation pathway, exerting antiviral responses against NDV. Guo et al. found that the Japanese encephalitis virus (JEV) induces apoptosis by inhibiting the STAT3-FOXO-BCL-6/p21 pathway (45). Our KEGG enrichment results showed that circRNAs in DEF cells post-NDV infection were enriched in apoptosis and apoptosis-related pathways, suggesting a similar antiviral mechanism in DEF cells, possibly by inducing apoptosis to resist NDVs infection. Further analysis revealed that caspase-8 and STAT1 genes, targeted by multiple significantly differentially expressed circRNAs, were widely involved in antiviral responses, growth inhibition, apoptosis, and other related biological processes (46, 47). These genes were mir-338-y-targeted, suggesting that the differentially expressed circRNAs might regulate gene expression mainly by affecting mir-338-y for antiviral responses against NDV infection. Mir-338-y was only targeted by circ-FBXW7 (novel_circ_000094), and circ-FBXW7 regulated multiple pathways in the KEGG enrichment, indicating that circ-FBXW7 may play an important role in antiviral responses to NDV infection in DEF cells.

Circ-FBXW7 overexpression inhibited NDV infection, which was promoted with circ-FBXW7 knockdown. Interestingly, this promotion and inhibition of virus replication can be seen at 12 h post-infection, since our sequencing results are based on the analysis of NDV infected samples at 9 h. Here, the circ-FBXW7 begins to function, hence significant differences were detected at 12 h post-infection. One round of NDV infection and replication takes about 6 h, indicating that circ-FBXW7 may not affect the invasion and replication stages of NDV virus. The *M* protein of NDV is thought to be dependent on ubiquitination to play the role of viral nucleocytoplasmic shuttling and release (48, 49). The circ-FBXW7-derived gene is thought to be involved in the regulation of ubiquitination (50). Therefore, we speculate that DEF cells may use circ-FBXW7 to regulate the ubiquitination modification process and affect the stability of *M* protein to achieve the effect of inhibiting NDV virus budding and release. Circ-FBXW7 has been widely studied, and its diverse functions have been suggested as cognate circRNA from circ-FBXW7 has distinct functions. Yang et al. demonstrated that FBXW7-185aa, encoded by the spanning junction open reading frame in circ-FBXW7 driven by an internal ribosome entry site, suppressed glioma tumorigenesis (51). Lu et al. showed that circ-FBXW7 regulates cancer cell generation and metastasis by inhibiting the NEK2 and mTOR signaling pathways and activating PTEN (52). Ye et al. demonstrated that circ-FBXW7 acts as a mir-197-3p sponge to inhibit the proliferation and migration of triple-negative breast cancer cells by increasing linear FBXW7 mRNA expression and inducing c-Myc degradation (53). Moreover, in a rectal cancer study, circ-FBXW7 was found in exosomes, which caused resistant cell sensitization to oxaliplatin, increased oxaliplatin-induced apoptosis, and inhibited oxaliplatin-induced epithelial-mesenchymal transition (54). These findings suggest that circ-FBXW7 exhibits diverse functions in different cancer processes; however, circ-FBXW7 exerting antiviral functions required elucidation. Therefore, to understand the interaction network between circ-FBXW7 and mir-338-y, KEGG enrichment analysis of mir-338-y-regulated target genes, mainly involved in cell cycle-related pathways, was performed. Cell cycle regulation plays an important role in cell growth and viral infection progression; thus, circ-FBXW7 may exert antiviral effects by targeting mir-338y to regulate cell cycle-related genes.

The circRNA produced by the virus during infection have also been extensively studied. Our study revealed that NDV infection of DEF cells altered circRNA expression profiles, while the significantly differentially expressed circRNA exerted different functions via multiple cellular processes and related pathways. Additionally, we investigated the production process of hosts to produce circRNA to combat viral infection. Circ-FBXW7 might have played a role in antiviral responses in DEF cells infected with NDV. However, the mechanism and related pathways remain to be determined. Finally, the formation and interaction mechanisms between viral circRNA and host circRNAs remain to be fully investigated. In addition, it is potential to study the regulation of innate immunity by NDV infection *in vivo* of waterfowl.

## Data availability statement

The datasets presented in this study can be found in online repositories. The names of the repository/repositories and accession number(s) can be found at: https://www.ncbi.nlm.nih.gov/geo/, GSE229335.

## Author contributions

LF participated in all the experiments and manuscript drafting. JR, YW, and YYC collected data and created the pictures. YCC assisted with the experiment. LC analyzed the experimental data. QL, ML, and CD reviewed the article. BX and TR helped to design the study. All authors contributed to the article and approved the submitted version.

## References

[1] Swayne DE. Diseases of poultry, 13th ed. Ames, IA: John Wiley & Sons. (2013).

[2] Chen L Song J Liu H Cai J Lin Q Xu C. Phylodynamic analyses of class I Newcastle disease virus isolated in China. Transbound Emerg Dis. (2021) 68:1294–304. doi: 10.1111/tbed.13785 32786140

[3] Xiang B Chen R Liang J Chen L Lin Q Sun M. Phylogeny, pathogenicity and transmissibility of a genotype XII Newcastle disease virus in chicken and goose. Transbound Emerg Dis. (2020) 67:159–70. doi: 10.1111/tbed.13335 31432620

[4] Otim OM Christensen H Mukiibi GM Bisgaard M. A preliminary study of the role of ducks in the transmission of Newcastle disease virus to in-contact rural free-range chickens. Trop Anim Health Prod. (2006) 38:285–9. doi: 10.1007/s11250-006-4309-4 17137130

[5] Bouzari M. The response of ducks to V4 Newcastle disease virus and its transmission to contact ducks and domestic chickens. Vet Res Forum. (2014) 5:145–8. PMID: 25568709 PMC4279625

[6] Kang Y Li Y Yuan R Li X Sun M Wang Z. Phylogenetic relationships and pathogenicity variation of two Newcastle disease viruses isolated from domestic ducks in southern China. Virol J. (2014) 11:147. doi: 10.1186/1743-422X-11-147 25117968 PMC4254411

[7] Meng C Rehman ZU Liu K Qiu X Tan L Sun Y. Potential of genotype VII Newcastle disease viruses to cause differential infections in chickens and ducks. Transbound Emerg Dis. (2018) 65:1851–62. doi: 10.1111/tbed.12965 30043428

[8] Kang Y Li Y Yuan R Feng M Xiang B Sun M. Host innate immune responses of ducks infected with Newcastle disease viruses of different pathogenicities. Front Microbiol. (2015) 6:1283. doi: 10.3389/fmicb.2015.01283 26635752 PMC4646970

[9] Elbestawy AR Ellakany HF El-Hamid H Zedan RE Gado AR Sedeik ME. Muscovy ducks infected with velogenic Newcastle disease virus (genotype VIId) act as carriers to infect in-contact chickens. Poult Sci. (2019) 98:4441–8. doi: 10.3382/ps/pez276 31111928

[10] Shao Q Xu W Yan L Liu J Rui L Xiao X. Function of duck RIG-I in induction of antiviral response against IBDV and avian influenza virus on chicken cells. Virus Res. (2014) 191:184–91. doi: 10.1016/j.virusres.2014.07.028 25128465

[11] Shao Q Xu W Guo Q Yan L Rui L Liu J. RIG-I from waterfowl and mammals differ in their abilities to induce antiviral responses against influenza a viruses. J Gen Virol. (2015) 96:277–87. doi: 10.1099/vir.0.069914-0 25371516

[12] Franz A Rabien A Stephan C Ralla B Fuchs S Jung K. Circular RNAs: a new class of biomarkers as a rising interest in laboratory medicine. Clin Chem Lab Med. (2018) 56:1992–2003. doi: 10.1515/cclm-2018-0231 29804099

[13] Dragomir M Calin GA. Circular RNAs in Cancer – lessons learned from microRNAs. Front Oncol. (2018) 8:179. doi: 10.3389/fonc.2018.00179 29911069 PMC5992376

[14] Kos A Dijkema R Arnberg AC van der Meide PH Schellekens H. The hepatitis delta (delta) virus possesses a circular RNA. Nature. (1986) 323:558–60. doi: 10.1038/323558a0 2429192

[15] Gao Z Li J Luo M Li H Chen Q Wang L. Characterization and cloning of grape circular RNAs identified the cold resistance-related Vv-circATS1. Plant Physiol. (2019) 180:966–85. doi: 10.1104/pp.18.01331 30962290 PMC6548266

[16] Shen M Li T Zhang G Wu P Chen F Lou Q. Dynamic expression and functional analysis of circRNA in granulosa cells during follicular development in chicken. BMC Genomics. (2019) 20:96. doi: 10.1186/s12864-019-5462-2 30700247 PMC6354403

[17] Rbbani G Nedoluzhko A Galindo-Villegas J Fernandes J. Function of circular RNAs in fish and their potential application as biomarkers. Int J Mol Sci. (2021) 22:7119. doi: 10.3390/ijms22137119 34281172 PMC8268770

[18] Zhang Y Zhang Y Li X Zhang M Lv K. Microarray analysis of circular RNA expression patterns in polarized macrophages. Int J Mol Med. (2017) 39:373–9. doi: 10.3892/ijmm.2017.2852 28075448 PMC5358696

[19] Salzman J Gawad C Wang PL Lacayo N Brown PO. Circular RNAs are the predominant transcript isoform from hundreds of human genes in diverse cell types. PLoS One. (2012) 7:e30733. doi: 10.1371/journal.pone.0030733 22319583 PMC3270023

[20] Chen X Yang T Wang W Xi W Zhang T Li Q. Circular RNAs in immune responses and immune diseases. Theranostics. (2019) 9:588–607. doi: 10.7150/thno.29678 30809295 PMC6376182

[21] Qu L Yi Z Shen Y Lin L Chen F Xu Y. Circular RNA vaccines against SARS-CoV-2 and emerging variants. Cells. (2022) 185:1728–1744.e16. doi: 10.1016/j.cell.2022.03.044 PMC8971115 35460644

[22] Li Y Zheng Q Bao C Li S Guo W Zhao J. Circular RNA is enriched and stable in exosomes: a promising biomarker for cancer diagnosis. Cell Res. (2015) 25:981–4. doi: 10.1038/cr.2015.82 26138677 PMC4528056

[23] Li X Liu CX Xue W Zhang Y Jiang S Yin QF. Coordinated circRNA biogenesis and function with NF90/NF110 in viral infection. Mol Cell. (2017) 67:214–227.e7. doi: 10.1016/j.molcel.2017.05.023 28625552

[24] Arora S Singh P Dohare R Jha R Ali SM. Unravelling host-pathogen interactions: ceRNA network in SARS-CoV-2 infection (COVID-19). Gene. (2020) 762:145057. doi: 10.1016/j.gene.2020.145057 32805314 PMC7428439

[25] Zhang X Yan Y Lei X Li A Zhang H Dai Z. Circular RNA alterations are involved in resistance to avian leukosis virus subgroup-J-induced tumor formation in chickens. Oncotarget. (2017) 8:34961–70. doi: 10.18632/oncotarget.16442 28415618 PMC5471026

[26] Wang ZY Guo ZD Li JM Zhao ZZ Fu YY Zhang CM. Genome-wide search for competing endogenous RNAs responsible for the effects induced by Ebola virus replication and transcription using a trVLP system. Front Cell Infect Microbiol. (2017) 7:479. doi: 10.3389/fcimb.2017.00479, PMID: 29209594 PMC5702449

[27] Shi J Hu N Li J Zeng Z Mo L Sun J. Unique expression signatures of circular RNAs in response to DNA tumor virus SV40 infection. Oncotarget. (2017) 8:98609–22. doi: 10.18632/oncotarget.21694 29228714 PMC5716754

[28] Sun M Xiang B Li Y Xie P Gao S Kang Y. Generation and evaluation of a genetically attenuated Newcastle disease virus rGM-VIIm as a genotype-matched vaccine. Virus Genes. (2017) 53:35–43. doi: 10.1007/s11262-016-1397-8 27718047

[29] Kang Y Feng M Zhao X Dai X Xiang B Gao P. Newcastle disease virus infection in chicken embryonic fibroblasts but not duck embryonic fibroblasts is associated with elevated host innate immune response. Virol J. (2016) 13:41. doi: 10.1186/s12985-016-0499-1 26975566 PMC4791923

[30] Li Y Xie P Sun M Xiang B Kang Y Gao P. S1PR1 expression correlates with inflammatory responses to Newcastle disease virus infection. Infect Genet Evol. (2016) 37:37–42. doi: 10.1016/j.meegid.2015.10.021 26597451

[31] Biacchesi S Skiadopoulos MH Yang L Murphy BR Collins PL Buchholz UJ. Rapid human metapneumovirus microneutralization assay based on green fluorescent protein expression. J Virol Methods. (2005) 128:192–7. doi: 10.1016/j.jviromet.2005.05.005 15955576

[32] Kong L You R Zhang D Yuan Q Xiang B Liang J. Infectious bronchitis virus infection increases pathogenicity of H9N2 avian influenza virus by inducing severe inflammatory response. Front Vet Sci. (2021) 8:824179. doi: 10.3389/fvets.2021.824179 35211536 PMC8860976

[33] Sun M Dong J Li L Lin Q Sun J Liu Z. Recombinant Newcastle disease virus (NDV) expressing duck Tembusu virus (DTMUV) pre-membrane and envelope proteins protects ducks against DTMUV and NDV challenge. Vet Microbiol. (2018) 218:60–9. doi: 10.1016/j.vetmic.2018.03.027 29685222 PMC7117350

[34] Rahman AU Habib M Shabbir MZ. Adaptation of Newcastle disease virus (NDV) in feral birds and their potential role in interspecies transmission. Open. Virol J. (2018) 12:52–68. doi: 10.2174/1874357901812010052 PMC6142666 30288195

[35] Kim LM King DJ Curry PE Suarez DL Swayne DE Stallknecht DE. Phylogenetic diversity among low-virulence Newcastle disease viruses from waterfowl and shorebirds and comparison of genotype distributions to those of poultry-origin isolates. J Virol. (2007) 81:12641–53. doi: 10.1128/JVI.00843-07 17855536 PMC2169019

[36] Liu H Wang Z Wang Y Sun C Zheng D Wu Y. Characterization of Newcastle disease virus isolated from waterfowl in China. Avian Dis. (2008) 52:150–5. doi: 10.1637/8030-061507-Reg 18459314

[37] Barber MR Aldridge JJ Webster RG Magor KE. Association of RIG-I with innate immunity of ducks to influenza. Proc Natl Acad Sci USA. (2010) 107:5913–8. doi: 10.1073/pnas.1001755107, PMID: 20308570 PMC2851864

[38] Wang L Liang W Wang S Wang Z Bai H Jiang Y. Circular RNA expression profiling reveals that circ-PLXNA1 functions in duck adipocyte differentiation. PLoS One. (2020) 15:e0236069. doi: 10.1371/journal.pone.0236069 32692763 PMC7373283

[39] Wu Y Xiao H Pi J Zhang H Pan A Pu Y. The circular RNA aplacirc_13267 upregulates duck granulosa cell apoptosis by the apla-miR-1-13/THBS1 signaling pathway. J Cell Physiol. (2020) 235:5750–63. doi: 10.1002/jcp.29509 31970783

[40] Li H Tang W Jin Y Dong W Yan Y Zhou J. Differential CircRNA expression profiles in PK-15 cells infected with pseudorabies virus type II. Virol Sin. (2021) 36:75–84. doi: 10.1007/s12250-020-00255-w 32617900 PMC7973350

[41] Zhao W Su J Wang N Zhao N Su S. Expression profiling and bioinformatics analysis of CircRNA in mice brain infected with rabies virus. Int J Mol Sci. (2021) 22:6537. doi: 10.3390/ijms22126537 34207166 PMC8234020

[42] Lu S Zhu N Guo W Wang X Li K Yan J. RNA-Seq revealed a circular RNA-microRNA-mRNA regulatory network in Hantaan virus infection. Front Cell Infect Microbiol. (2020) 10:97. doi: 10.3389/fcimb.2020.00097, PMID: 32232013 PMC7083127

[43] Guo L Mu Z Nie F Chang X Duan H Li H. Thymic transcriptome analysis after Newcastle disease virus inoculation in chickens and the influence of host small RNAs on NDV replication. Sci Rep. (2021) 11:10270. doi: 10.1038/s41598-021-89464-1, PMID: 33986327 PMC8119446

[44] Xue B Yang D Wang J Xu Y Wang X Qin Y. ISG12a restricts hepatitis C virus infection through the ubiquitination-dependent degradation pathway. J Virol. (2016) 90:6832–45. doi: 10.1128/JVI.00352-16 27194766 PMC4944290

[45] Guo F Yu X Xu A Xu J Wang Q Guo Y. Japanese encephalitis virus induces apoptosis by inhibiting Foxo signaling pathway. Vet Microbiol. (2018) 220:73–82. doi: 10.1016/j.vetmic.2018.05.008 29885805

[46] Fritsch M Gunther SD Schwarzer R Albert MC Schorn F Werthenbach JP. Caspase-8 is the molecular switch for apoptosis, necroptosis and pyroptosis. Nature. (2019) 575:683–7. doi: 10.1038/s41586-019-1770-6 31748744

[47] Verhoeven Y Tilborghs S Jacobs J De Waele J Quatannens D Deben C. The potential and controversy of targeting STAT family members in cancer. Semin Cancer Biol. (2020) 60:41–56. doi: 10.1016/j.semcancer.2019.10.002 31605750

[48] Tan L Zhang Y Qiao C Yuan Y Sun Y Qiu X. NDV entry into dendritic cells through macropinocytosis and suppression of T lymphocyte proliferation. Virology. (2018) 518:126–35. doi: 10.1016/j.virol.2018.02.011 29481983

[49] Peng T Qiu X Tan L Yu S Yang B Dai J. Ubiquitination on lysine 247 of Newcastle disease virus matrix protein enhances viral replication and virulence by driving nuclear-cytoplasmic trafficking. J Virol. (2022) 96:e0162921. doi: 10.1128/JVI.01629-21 34705566 PMC8791254

[50] Chakravorty D Ghosh A Saha S. Computational approach to target USP28 for regulating Myc. Comput Biol Chem. (2020) 85:107208. doi: 10.1016/j.compbiolchem.2020.107208, PMID: 32028107

[51] Yang Y Gao X Zhang M Yan S Sun C Xiao F. Novel role of FBXW7 circular RNA in repressing glioma tumorigenesis. J Natl Cancer Inst. (2018) 110:304–15. doi: 10.1093/jnci/djx166 28903484 PMC6019044

[52] Lu H Yao B Wen X Jia B. FBXW7 circular RNA regulates proliferation, migration and invasion of colorectal carcinoma through NEK2, mTOR, and PTEN signaling pathways in vitro and in vivo. BMC Cancer. (2019) 19:918. doi: 10.1186/s12885-019-6028-z 31519156 PMC6744671

[53] Ye F Gao G Zou Y Zheng S Zhang L Ou X. circFBXW7 inhibits malignant progression by sponging miR-197-3p and encoding a 185-aa protein in triple-negative breast Cancer. Mol Ther Nucleic Acids. (2019) 18:88–98. doi: 10.1016/j.omtn.2019.07.023, PMID: 31536884 PMC6796723

[54] Xu Y Qiu A Peng F Tan X Wang J Gong X. Exosomal transfer of circular RNA FBXW7 ameliorates the chemoresistance to oxaliplatin in colorectal cancer by sponging miR-18b-5p. Neoplasma. (2021) 68:108–18. doi: 10.4149/neo_2020_200417N414 33147048

