# Supplementary figures and images for: Circular RNAs are associated with the resistance to Newcastle disease virus infection in duck cells
Source: Front Vet Sci. 2023 Sep 29;10:1181916. doi: 10.3389/fvets.2023.1181916 (PMC10570413; doi:10.3389/fvets.2023.1181916)

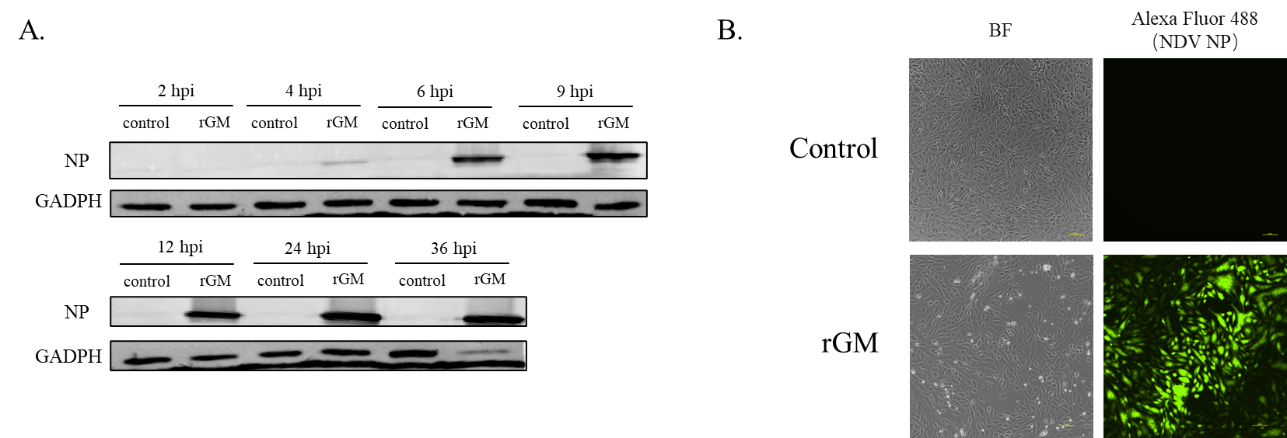

Supplement: Supplementary file 2 [file Image_1.TIF]
